# A prospective clinical trial of diathermy ablation for patients with high-grade cervical intraepithelial neoplasia from a single institution in Japan

**DOI:** 10.1038/s41598-024-53197-8

**Published:** 2024-02-01

**Authors:** Takeji Mitani, Iwao Kukimoto, Tetsuya Tsukamoto, Hiroyuki Nomura, Takuma Fujii

**Affiliations:** 1 Department of Obstetrics and Gynecology, Fujita Health University, School of Medicine, 1-98, Dengakugakubo, Toyoake, Aichi 470-1192 Japan; 2 Department of Gynecology, Fujita Health University, School of Medicine, 1-98, Dengakugakubo, Toyoake, Aichi 470-1192 Japan; 3 Department of Pathology, Fujita Health University, School of Medicine, 1-98, Dengakugakubo, Toyoake, Aichi 470-1192 Japan; 4 Department of Gynecology, Fujita Health University Okazaki Medical Center, 1, Gotanda, Harisaki-cho, Okazaki, Aichi 444-0827 Japan; 5 Pathogen Genomics Center, National Institute of Infectious Diseases, Musashi-Murayama, 4-7-1, Gakuen, Tokyo, 208-0011 Japan

**Keywords:** Surgical oncology, Preventive medicine, Cancer prevention, Human papilloma virus

## Abstract

Approximately 500,000 women are diagnosed with cervical cancer annually, with high-grade cervical intraepithelial neoplasia (CIN) estimated to be 20 times higher. The diathermy ablation is an inexpensive minimally invasive surgeries for CIN. However, little is known about the treatment outcomes. A prospective clinical trial was therefore conducted to evaluate ablation outcomes based on detailed colposcopy findings, cytology, and biopsy results over a two-year period. We enrolled CIN2 (n = 32) and CIN3 (n = 7) patients. Eligibility criteria included: aged between 29 and 49 (median: 36, mean: 36.3), visible transformation zone with high-grade lesions not entirely occupying the cervix, and histologically diagnosed with CIN2 or CIN3. Cytology and HPV genotyping were performed, and colposcopic findings were evaluated. Colposcopy-guided diathermy ablation was conducted by a certified gynecologic oncologist. The incidence of recurrent or residual disease was 5.1% (2/39, 95% confidence interval: − 0.02 to 0.12). The prevalence of HPV infection at 12 months decreased after surgery, as 67.6% (23/34, 0.52–0.83) of patients were HPV-negative. No severe adverse events were reported, while there were five pregnancies with full-term deliveries. The promising outcome was possibly due to selection of rigorous surgical indication and skilled surgical techniques. The study highlights the importance of experienced and skilled colposcopists.

*TrialRegistry* This study was registered in the clinical trial registration system of the University hospital Medical Information Network Clinical Trials Registry (UMIN-CTR ID: UMIN000024483). Open for the trial to the public through the website: 01/11/2016. First registration of the patient: 30/01/2017.

## Introduction

Each year worldwide, approximately 500,000 women are diagnosed with cervical cancer. The number of patients with high-grade cervical intraepithelial neoplasia (CIN), which is a precancerous lesion of the cervix, is estimated to be 20 times higher^1^. In England, for example, 2,700 women are treated for cervical cancer whereas over 60,000 are treated for CIN each year^2^. A minimally invasive method of preventing invasive cancer by removing the lesion through local destruction or ablation of the cervix has become a popular method. This method has advantages in terms of fertility preservation, especially in younger women. Large loop excision of the transformation zone (LLETZ) or the loop electrical excision procedure (LEEP) have become the standard treatments in developed countries^3^. However, diathermy, thermal ablation, cryotherapy and laser vaporization are less minimally invasive but potentially curative alternative methods. The advantage of these latter methods is that they avoid the risk of preterm delivery; patients in their 20s to early 40s who wish to preserve their fertility may prefer these methods^4^. Thermal ablation is easy to learn how to use the device and does not require expensive equipment, and is therefore popular in developing countries^3,5^. However, the ablation device is not approved for use in Japan. In reference hospitals, laser vaporization is often the preferred choice over cryotherapy, although the equipment is expensive and is thus not widely used in small or medium-sized hospitals or clinics. In contrast, the equipment required for diathermy ablation is inexpensive and can be easily operated by the smaller facilities.

Local therapy for high- grade CIN has a recurrence or residual rate of approximately 10–20% at the two-year follow up term^3,6–11^. LEEP is advantageous in that it allows a clear pathological diagnosis, but the procedure is associated with poorer obstetric outcomes. In contrast, ablation is less invasive but may be associated with a high recurrence rate, possibly due to changes of the structure of the cervix between LEEP and ablation was different^12^. Because ablation is a good treatment choice for smaller or less severe cervical lesions, we believe that further reducing the risk of obstetric complications through rigorous selection criteria would have a significant positive impact in the clinic. For the purpose, combining detailed colposcopy findings with cytology and biopsy results will be required to make this a reality. In this study, we conducted a prospective clinical trial under the eligibility criteria for ablation, and evaluated outcomes over a two-year follow-up period.

## Materials and methods

### Trial design

The primary endpoint was to assess the residual or recurrence rate of CIN2 or CIN3 lesions over a 24-month post-diathermy ablation period. The secondary endpoint was to assess the suitability of HPV testing for prediction of residual or recurrent disease.

### Inclusion and exclusion criteria of the patients

Eligibility criteria of the patients were as follows.

1. Aged between 20 and 50 at the time of consent. Considering that the average age of menopause in Japanese women is 51 years old, and the squamo-columnar junction of the cervix becomes invisible after menopause, the upper limit is set at 50 years old.
2. Transformation zone (TZ) was either entirely or partially visible (TZ1 or TZ2, respectively)^13^, and colposcopic analysis indicated that high- grade abnormal lesions did not entirely occupy the cervix. Patients with low grade lesions that occupied the entire cervix were still deemed eligible. The patients must have been diagnosed with CIN2 or CIN3 by histology, and must have been free of concomitant glandular lesions as evidenced by colposcopic-directed biopsy. Eligible cytology classifications were NILM, ASC-US, LSIL, ASC-H, AGC or HSIL.
3. Performance status 0 or 1

The exclusion criteria for registration were as follows:

1. Previous treatment for cervical tumor such as hysterectomy, cone resection, ablation, or radiation therapy.
2. Patients with active multiple cancers that are being treated.
3. Patients with psychosis or psychiatric symptoms that would make it difficult for them to participate in the study.
4. Patients who were pregnant or were HIV-positive.

### Trial conduct and oversight

The number of patients to be enrolled was determined by considering the number of visits by patients with the disease, the number of patients expected to be able to be followed up for two years after treatment, and the number of personnel required to follow up the patients. Colposcopy video imaging system combined with OCS-500 and VISERA PRO OTV-S7Pro. (Olympus Corporation, Tokyo, Japan) was utilized for colposcopy. Colposcopies were conducted with application of 3% acetic acid, but the Shiller test was not used. The potential bias was due to that the patients recruitment, colposcopy, surgery, and follow-up were performed by a certified oncologist.

**Figure 1:**
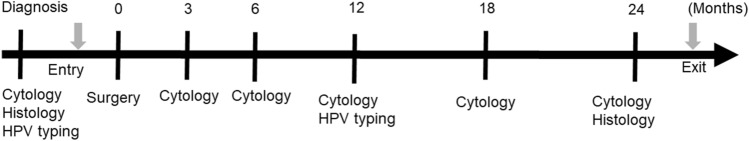
Time course of the clinical trial for collection of the specimens. After surgery, LBC was collected at 3, 6, 12, 18 and 24 months. HPV genotyping was conducted before and 12 months after surgery. At 24 months, colposcopy-directed punch biopsy was conducted for histology.

Patients underwent ablation within three months of histological diagnosis at the outpatient ward. Surgery dates were planned to avoid the days during which patients had their menstrual period. The colposcopy-guided diathermy ablation (Sabre Genesis™, CONMED NY, USA) was conducted under the local anesthesia with 1% lidocaine, with a preceding excision for another biopsy to confirm the absence of invasion or adenocarcinoma in situ (AIS). The ablation was conducted using a ball electrode under the colposcope. After the ablation of the worst lesion, the squamocolumnar junction was fulgurated circumferentially. The power of the coagulation mode was 30W. The surgery took approximately 10 min or less (including the time required for local anesthesia to take effect). Assessments of the procedure’s safety was based on whether any major complications, including hospitalization and disability, arose. Patients were advised to have an outpatient visit at 3, 6, 12, 18 and 24 months after surgery (Fig. 1). Patients who did not come to the hospital were contacted by telephone and encouraged to make an appointment. Even if a patient did not make an outpatient visit during follow-up and data were missing at a certain point during the observation period, if the patient came to the hospital at the next observation point, the data were used at that point for subsequent analysis. Patients were assessed by cytology and physician-led HPV testing unless abnormal results were obtained. Biopsy after surgery was postponed until exit (24 months after surgery) unless the cytology results or colposcopy findings were suggestive of recurrent or residual disease. Regardless of the findings of colposcopy, a biopsy was performed at 24 months after surgery as an exit test. If patients were diagnosed with CIN2 or worse at the time of followed-up, we defined residual disease as detection of dysplastic cells in the first 6 months post-surgery, whereas recurrence was defined by appearance of disease between 6 and 24 months. The data were collected for surveying adverse events and obstetric outcomes until November 2021. Patients were informed of the final histologic diagnosis and advised that the clinical trial was over, but that they should continue to follow-up. Monitoring audits were conducted by TS, confirming the absence of protocol violations and serious complications in the clinical trial.

### Cytology and HPV genotyping

**Figure 2:**
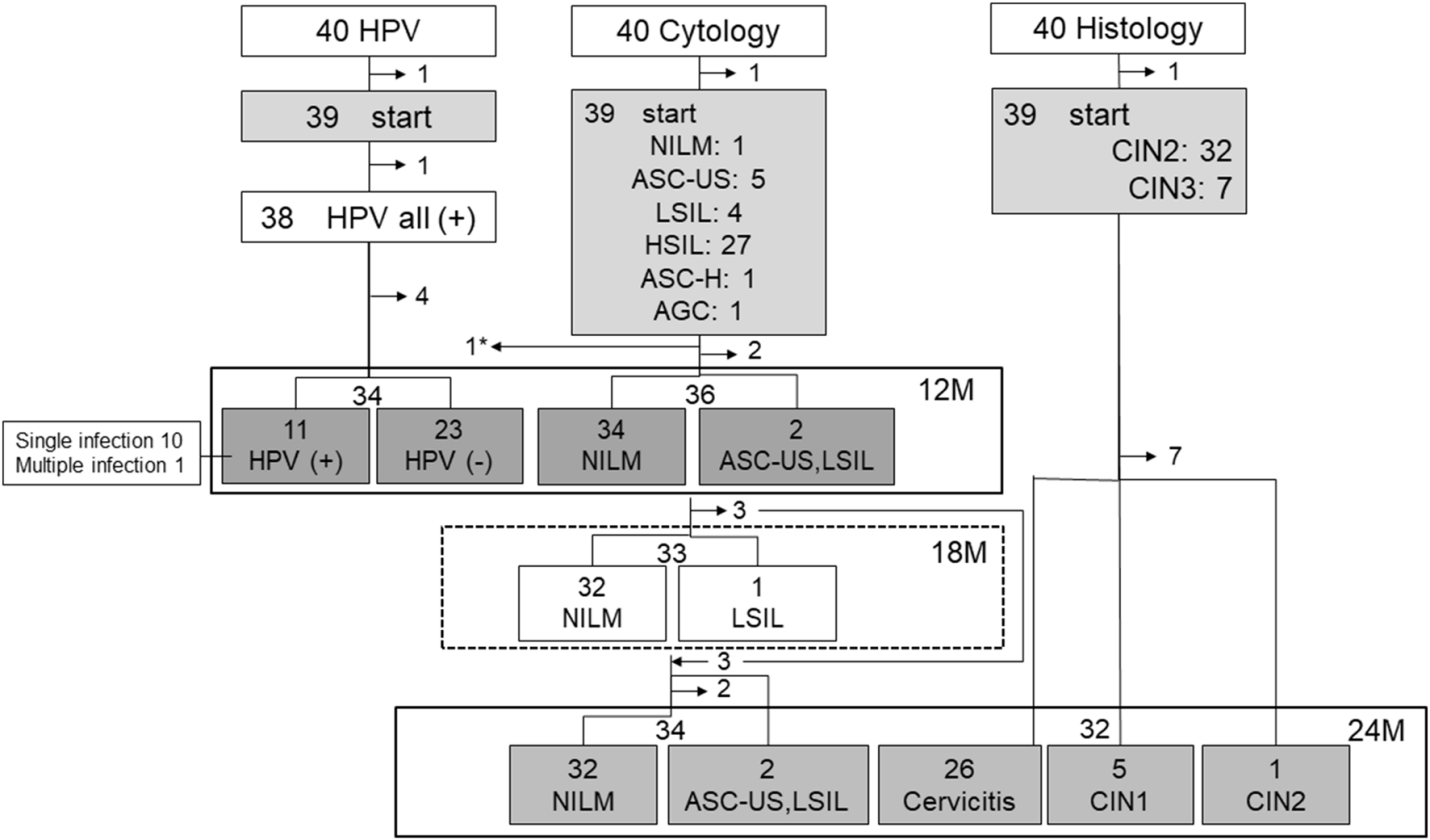
Outcome of enrolled patients through 24 months postoperatively. 1*: one patient had HSIL at 3 and 6 months after surgery. As she had residual disease, this patient underwent ablation again at 6 months.

Specimens were periodically collected for liquid-based cytology (LBC) and HPV genotypes (Fig. 2). Cytological interpretation was classified according to the Bethesda 2001 system. HPV genotype assays were performed using a polymerase chain reaction with PGMY primers followed by reverse line blot hybridization^14^. This assay can detect 31 HPV genotypes, including HPV 6, 11, 16, 18, 26, 31, 33, 34, 35, 39, 40, 42, 44, 45, 51, 52, 53, 54, 55, 56, 57, 58, 59, 66, 68, 69, 70, 73, 82, 83, and 84.

### Evaluation of the colposcopic findings

Factors including normal/abnormal findings, TZ type, the size of the abnormal lesion, and interpretation of CIN grade were identified based on the colposcopy findings^13^.

### Statement of ethics

All procedures performed in studies involving human participants were in accordance with the ethical standards of the Institutional Research Committee and with the 1964 Helsinki Declaration and its later amendments or comparable ethical standards. The study protocol was approved by the ethical committee of Fujita Health University (HM20-096). Written informed consent was obtained from each patient.

## Results

### Demographic and clinical characteristics of patients

Forty patients were enrolled in the outpatient clinic of Fujita Health University, Aichi, Japan from November 2016 through August 2019. Among 40 patients, one patient was excluded from the analysis because she declined to participate. The 39 patients with CIN2 (n = 32) or CIN3 (n = 7), who met the eligibility criteria were followed up for 2 years, except for those who did not respond to visits (Table 1). Enrolled patients were 29–49 years of age (median: 36, mean: 36.3). The rate of smoking history including current smoking was 14/39 (35.9%, 95% CI 0.21–0.51). The Brinkman index was 100 (median) in the smoking group. The combination of preoperative cytology and histology was as follows; HSIL/CIN2: 22/39 (56.4%, 95% CI 0.41–0.72), HSIL/CIN3: 5/39 (12.8%, 95% CI 0.02–0.23), LSIL/CIN2: 4/39 (10.2%, 95% CI 0.01–0.2), ASC-US/CIN2: 5/39 (12.8%, 95% CI 0.02–0.23), ASC-H/CIN3: 1/39 (2.5%, 95% CI − 0.02 to 0.07), AGC/CIN2: 1/39 (2.5%, 95% CI − 0.02 to 0.07), NILM /CIN2: 1/39 (2.5%, 95% CI − 0.02 to 0.07). In cases for which there was a slight discrepancy between cytology and histology, the indication for surgery was determined based on the cytological and colposcopy findings. Cases with significant discrepancies were not enrolled in this study.

**Table 1:** Patient characteristics (N = 39).

Age	29–49 (median 36)	
Gravity	0–4 (median 2)	
Parity	0–3 (median 1)	
Smoking History	14/39 (35.9%)	
	Brickman index in positive group 100 (median), 136 (mean)	
Preoperative cytology	NILM	1 (2.6%)
	ASC-US	5 (12.8%)
	LSIL	4 (10.3%)
	ASC-H	1 (2.6%)
	HSIL	27 (69.2%)
	AGC	1 (2.6%)
Preoperative histology	CIN2	32 (82.1%)
	CIN3	7 (17.9%)
Status of HPV Infections (before surgery)	Single	26 (66.6%)
	Multiple	12 (30.8%)
	ND	1 (2.6%)

### Reconfirm biopsy at the time of surgery

The patients were diagnosed by histological analysis before they were placed on the clinical trial. Reconfirm biopsy performed at the time of surgery excluded the presence of microinvasion or AIS in all patients. The area of the second biopsy was 4.7 mm^2^ (median). Since this is such a small amount of tissue, it is unlikely to have affected the ablation procedure.

### Time course of the change of cytology and histology

Thirty-eight of 39 (97.4%, 95% CI 0.92–1.02) patients had abnormal cytology before surgery. At 12, 18 and 24 months after surgery, 2 of 36 (5.6%, 95% CI − 0.02 to 0.13), 1 of 33 (3.0%, 95% CI − 0.03 to 0.09), and 2 of 34 (5.8%, 95% CI − 0.02 to 0.14) patients had abnormal cytological results, respectively (Fig. 2). For all patients except patient No. 29 (Table S3), no cytological findings suggestive of recurrent or residual high-grade intraepithelial lesions (HSIL) were observed up to 18 months postoperatively. Consequently, colposcopy was not performed except No.29 during this period. No biopsies were taken from any patients until the18-month follow-up period. Patient no. 29 exhibited HSIL at 3 months postoperatively, and a repeat cytology at 6 months postoperatively showed HSIL again. In this point, colposcopy revealed suspicious findings of residual lesions, prompting a repeat ablation. One patient had CIN2 and five had CIN1 at exit (24 months after surgery). Overall, 2 of 39 patients (5.1%, 95% CI − 0.02 to 0.12) were classified as having residual (no.29) or recurrent disease (no.23) during the study period, since we did not consider CIN1 to be a recurrence of a precursor of cervical cancer.

### The status of HPV infection before surgery and 12 months after surgery

Before surgery, all patients who had an HPV test were HPV-positive. Twenty-six of 39 (66.6%, 95% CI 0.52–0.81) HPV-positive patients had single infections. Before surgery, 11of 12 patients with CIN2 had multiple HPV infections. In contrast, six of seven patients with CIN3 had a single HPV infection. In the cases of single infection, HPV16, − 31, − 35, − 51, − 52, − 58 or − 66 was detected before surgery and HPV16, − 39, − 51, − 52, − 53, − 56, − 58 or 81 after surgery. In multiple infections, HPV 16, − 31, − 33, − 39, − 44, − 51, − 52, − 53, − 54, − 56, − 58, − 59, − 66, − 68, − 69 and − 82 were detected before surgery and HPV74 and -82 after surgery. After surgery, the 34 patients were either HPV-negative (23; 67.6%, 95% CI 0.52–0.83), or had single (10; 29.4%, 95% CI 0.14–0.45) or multiple infections (1;2.9%, 95% CI − 0.03 to 0.09).

### Association between HPV and cytology

Twelve months after surgery, 34 of 36 patients (94.4%, 95% CI 0.87–1.02) were diagnosed with NILM by cytology, and nine of these patients with NILM (26.5%, 95% CI 0.12–0.41) were HPV-positive (Fig. 2 and Table 2). Of the 11 HPV-positive patients, eight had the same persistent infection from the preoperative period and three had a change in type. When analyzed from the viewpoint of whether or not HPV infection can be detected by cytology, nine of 11 cases were NILM, indicating that the sensitivity of HPV detection by cytology is low. On the other hand, at 12 months, the HPV-positive rate among NILM patients was 26.4% (9/34, 95% CI 0.12–0.41).

**Table 2:** Association between cytology and HPV infections at 12 months after surgery (N = 11).

Cytology	HPV positive
NILM	9 (67.6%)
ASC-US	1 (29.4%)
LSIL	1 (2.9%)

### Combination of histology, cytology, HPV and colposcopic findings 24 months after surgery

At 24 months, there were 5 patients with CIN1 (Patient ID.1, 6, 10, 11, 15) and one with CIN2 (Patient ID.23). All patients with CIN1/ 2 at 24 months had NILM at 12 and 18 months; one with CIN1 was HPV39-positive at 12 months, whereas the other four were HPV-negative. Though 11 patients were HPV-positive at 12 months, only one HPV58-positive patient had CIN2 at 24 months, suggesting that neither HPV positivity nor cytology was able to predict recurrence. However, none of the patients who were HPV-negative after surgery had recurrent CIN2 or worse in this period. The colposcopic findings at 24 months were normal in four of five patients, suggesting that colposcopy after surgery was relatively ineffective unless the colposcopic findings suggested high- grade CIN.

### Evaluation of colposcopic findings including TZ type, the size of the abnormal lesion and interpretation of CIN grade

The colposcopic data at entry revealed abnormalities in 38/39 (97.4%, 95% CI 0.92–1.02) of patients. One patient classified as normal (squamous metaplasia) by colposcopy was diagnosed as LSIL following cytology, and was positive for both HPV56 and HPV82; the biopsy indicated CIN2 in this case. We have recruited 36/39 (92.3%, 95% CI 0.84–1.01) patients with TZ1 (Table 3). The expected grades of CIN based on colposcopic findings were classified into 6 categories: Metaplasia, Low, Low/Moderate, Moderate, Moderate/High and High. Since the definition of ‘moderate dysplasia’ is ambiguous even in histological analysis, it was difficult to determine strict criteria for moderate dysplasia by colposcopy. Indeed, 31 of 39 patients (79.5%, 95% CI 0.67–0.92) were placed into one of the three ‘Moderate’ categories (Table S2). Of note, the percentage of the high- grade lesion of the cervix was less than 50% with a median of 25% in all patients (Table 4). The rate of visible TZ at 24 months after surgery was still 69% (22/32, 95% CI 0.53–0.85; Table 3), suggesting that ablation was less invasive. However, postoperative changes made it difficult to interpret the colposcopic findings. Of note, the colposcopic impression at 24 months was not consistent with the histology results.

**Table 3:** Transformation zone (TZ) type and colposcopic findings.

Type	Number of patients	
	0 M	24 M
TZ		
TZ1	36	6
TZ2	3	13
TZ3	0	3
No visible squamo-columnar junction	0	10
ND	0	7
Impression		
Normal	1*	17
Abnormal	38	5
ND	0	17

*LSIL in cytology, HPV56 and HPV82 positive.

**Table 4:** The size of the high-grade lesion as percentage of cervix at 0 M.

Rate	Number of patients
< 25%	20
25% ≥ , ≤ 50%	19
Mean	28.6% (0–50)
Median	25%

The rate of the abnormal lesions in the cervix, e.g., 0%; normal, 100%; entire of the cervix.

### Association of TZ type and residual or recurrent disease

Two of three patients with TZ2 had a residual or recurrent disease after surgery (Fig. S1). One patient with a residual lesion underwent a second ablation 6 months after surgery. Another was diagnosed with CIN2 as a recurrent disease at 24 months.

### Adverse events and obstetric outcomes

No serious complications including cervical stenosis were reported during the study period. There were no cases requiring postoperative hospitalization for any reason, and there were no unscheduled outpatient visits reporting postoperative bleeding, pain, or other complaints.　Five patients had pregnancies and full-term deliveries.

## Discussion

Here we report what we believe is the first Japanese prospective clinical trial of diathermy ablation for CIN2/3. There is considerable inter-physician variability with regard to the diagnosis of CIN2^15,16^ Therefore, one recent proposal is to ‘watch and wait’ prior to making a recommendation for surgery^17,18^. All participants in this study were therefore informed and assured that CIN can resolve spontaneously. We found that the rate of recurrent or residual disease (our primary endpoint) was 5.1% (2/39) over the two-year period. A reason for the lower rate of recurrent or residual disease compared to previous papers is the rigorous indication for surgery. In this study, the colposcopy and cytological findings, as well as the biopsy results and age, were taken into consideration when determining patient eligibility. Although previous articles on local CIN therapy mentioned the importance of colposcopy findings^19^, no article has described such results in detail. This may be because it is difficult to describe colposcopy findings in a written form. To address this issue, we analyzed colposcopic findings in individual patients. We would like to reiterate the importance of congruent results from cytology, histology, and colposcopy, and the requirement for abnormal lesions to be localized^19^. If the target lesion is large, there is a possibility of microinvasion^20^, and residual or persistent disease may manifest. Therefore, the size of the occupied abnormal lesion is vital for the selection of the patients for ablation (Table 4). In the criteria for surgical indications, the high-grade abnormal lesions did not entirely occupy the cervix, but in all cases, this proportion did not exceed 50%, with a median of 25%. The fact that the recurrence and residual rate was 5.1% in the current treatment outcomes suggests that patient selection in the indication of surgery may be extremely crucial. Visualization of the TZ, the target of the ablation, is also an essential requirement. In an earlier report, endocervical curettage was not used as a criterion for mandatory assignment to ablation treatment^10^. We did not perform endocervical curettage. Since three of the registered patients (7.6%) had TZ2, and two of them had recurrent or residual lesions (Table 3, Fig. S1), we suggest that patients with TZ2 type should be excluded from indication of future ablation cohorts at our institution. No severe adverse events were reported that interfered with daily life. Although no visible squamo-columnar junction was observed in 10/32 (31.3%) of patients 24 months post-surgery^21^, no cervical stenosis was observed (Table 3). In cone resection, resection heights longer than 10 mm carry a higher risk of preterm delivery^22–24^. In contrast, ablation does not appear to be related to preterm delivery^25^; this is consistent with our finding that five patients treated with ablation had full term pregnancies.

The HPV detection rate after treatment is about 30%^21,26^, and our data are consistent with this rate. As for HPV positivity, we do not know whether it continues to persist or is reacquired despite disappearing after treatment. HPV may still be present in the vaginal mucosa after treatment, and this could be a source of reinfection. Positive and negative results depend on the amount of virus and the HPV detection method, so care must be taken in interpreting the results. Approximately one-third of patients were infected with multiple HPV types before treatment. At the 12-month time point after surgery, with one exception (ID: 2), all HPV-positive patients were infected with a single genotype. The secondary endpoint of this study was to assess the ability of HPV testing for prediction of residual or recurrent disease. Patients that were HPV-negative at 12 months post-surgery have not experienced recurrence.

This article has the following strengths. The colposcopists are not only required to have the skill to target the worst lesion for biopsy, but must also to be able to properly evaluate the extent and degree of the lesion, to understand cytology, HPV testing, and histology in an integrated manner, to decide whether a cone resection or ablation is the best approach, and to treat properly^27^. In this study, diagnosis and treatment were performed by a certified colposcopist. Proper selection of patients as well as the skills of the surgeon, have a significant influence on the outcome^10^. The HPV test we adapted was performed using a method that is considered the global standard^14,28^. Efforts were made to exclude the possibility of invasive cancer or AIS by performing another biopsy at the time of ablation. The diathermy ablation is easily performed in less than 10 min on an outpatient basis. The equipment is inexpensive and easy to prepare at any hospital or clinic^29^. Our diathermy ablation offers the advantage that surgeons can treat the area based on the extent and severity of each patient's lesion. Conversely, a potential drawback may be the variation in surgeon’s skills, particularly in capturing the lesion. Although the commercially available thermal ablation equipment has the advantage of minimizing differences in technique between surgeons, it is a uniform treatment, making it difficult to respond to individual differences. This paper evaluates the skills of colposcopists from the perspective of therapeutic intervention. Colposcopists play a role in both diagnosis and treatment, but evaluating their proficiency is challenging, and not every gynecologic oncologist is necessarily a colposcopist. While the reproducibility and universality of surgical outcomes pose challenges, the merit of this paper lies in demonstrating that with proficiency as a colposcopist, such treatment can be considered as one of the options. In the future, the cultivation of highly skilled colposcopists is deemed crucial.

The disadvantages of this study include the small number of patients enrolled because it was performed by a single physician and the short observation period of two years. If multiple physicians are conducted, the sample size can be increased, but interoperator bias in diagnostic treatment becomes a problem. In this clinical trial, we limited the enrollment to one surgeon and only those within that surgeon's handling capacity. Based on the results of this trial, we believe that the next trial will need to be validated with multiple physicians in multiple institutions. It was also difficult to conduct a follow-up study for more than two years because of personnel and insurance costs required for a prospective clinical study. Because HPV testing was done only once after the surgery, we prefer to determine HPV status at 24 months in the following studies. The long-term prognosis of ablation or LEEP is unclear, despite reports that 80% of recurrences occur within 2 years^6,10^. In the 2-year postoperative period, CIN was found on biopsy even in the absence of abnormal cytology or colposcopy findings. Given the limitations of colposcopy and cytology, it may be necessary to use HPV testing or other methods as a triage. Since recurrence may manifest more than 2 years post-surgery, clinicians should continue to examine whether recurrence correlates with HPV persistence.

Smoking is a risk factor that can reduce the chances of spontaneous CIN regression. In a previous cohort study, half of Japanese patients with CIN were current or past smokers^30^. Together with our findings that smoking rates were also high among patients, the dangers of smoking as a risk factor for CIN and cervical cancer should be conveyed to the general public.

In conclusion, diathermy equipment is inexpensive and convenient, and diathermy ablation is a simple procedure that can be performed rapidly in an outpatient clinic. It is important to train experienced colposcopists and–based on the results of this study–the impact of ablation on long-term prognosis should continue to be reported by the global network of cervical cancer physicians.

### Supplementary Information

Supplementary Information.

## Data Availability

Additional data has been provided in the electronic supplementary material available for download.
