# Supplementary material for: A prospective clinical trial of diathermy ablation for patients with high-grade cervical intraepithelial neoplasia from a single institution in Japan
Source: Sci Rep. 2024 Feb 1;14:2632. doi: 10.1038/s41598-024-53197-8 (PMC10834401; doi:10.1038/s41598-024-53197-8)

Figure S1 Residual / recurrent cases before and after ablation

A patient (ID.23) was diagnosed with CIN2 at 24 months after surgery. After application of acetic acid, the white lesion extended to the OS canal before surgery (A). The abnormal lesion was observed in the canal at 24 months after surgery (B).

Another patient (ID.29) was diagnosed with residual disease after 3 months. After application of acetic acid, white lesion extended to the OS canal before surgery (C). The abnormal lesion was observed in the posterior position of the OS after surgery (D).

Table S1. Identification of HPV genotypes

| Before surgery (N=53) | | |  | After surgery at 12 months (N=35) | | |
| --- | --- | --- | --- | --- | --- | --- |
| HPV | 16 | 15 |  | HPV | 16 | 1 |
|  | 31 | 3 |  |  | 31 | 1 |
|  | 35 | 1 |  |  | 35 | 0 |
|  | 39 | 1 |  |  | 39 | 1 |
|  | 44 | 1 |  |  | 44 | 0 |
|  | 51 | 5 |  |  | 51 | 1 |
|  | 52 | 9 |  |  | 52 | 1 |
|  | 53 | 1 |  |  | 53 | 1 |
|  | 54 | 2 |  |  | 54 | 0 |
|  | 56 | 2 |  |  | 56 | 1 |
|  | 58 | 7 |  |  | 58 | 2 |
|  | 59 | 1 |  |  | 59 | 0 |
|  | 66 | 2 |  |  | 66 | 0 |
|  | 68 | 1 |  |  | 68 | 0 |
|  | 69 | 1 |  |  | 69 | 0 |
|  | 74 | 0 |  |  | 74 | 1 |
|  | 81 | 0 |  |  | 81 | 1 |
|  | 82 | 1 |  |  | 82 | 1 |
| Negative | | 0 |  | Negative | | 23 |

Footnotes: This assay can detect 31 HPV genotypes, including HPV 6, 11, 16, 18, 26, 31, 33, 34, 35, 39, 40, 42, 44, 45, 51, 52, 53, 54, 55, 56, 57, 58, 59, 66, 68, 69, 70, 73, 82, 83, and 84. Methods more in detail in materials and methods.

Table S2　Classification of the colposcopic findings

|  | Colposcopic findings | Normal |  | Abnormal | | | | |
| --- | --- | --- | --- | --- | --- | --- | --- | --- |
|  |  |  |  |  | | | | |
|  |  |  |  | Low | Low  /Moderate | Moderate | Moderate  /High | High |
| Number of patients | 0M (before surgery) | 1 |  | 2 | 6 | 21 | 4 | 5 |
|  | 24M after surgery | 17 |  | 3 | 0 | 2 | 0 | 0 |

Footnotes: This is an indication of the degree of CIN that the colposcopy findings are expected to have. Abnormal findings low are classified as CIN1 in histology, moderate as CIN2, and high as CIN3. Since borderline regions exist, those corresponding to the respective borderline regions are also added to the table.


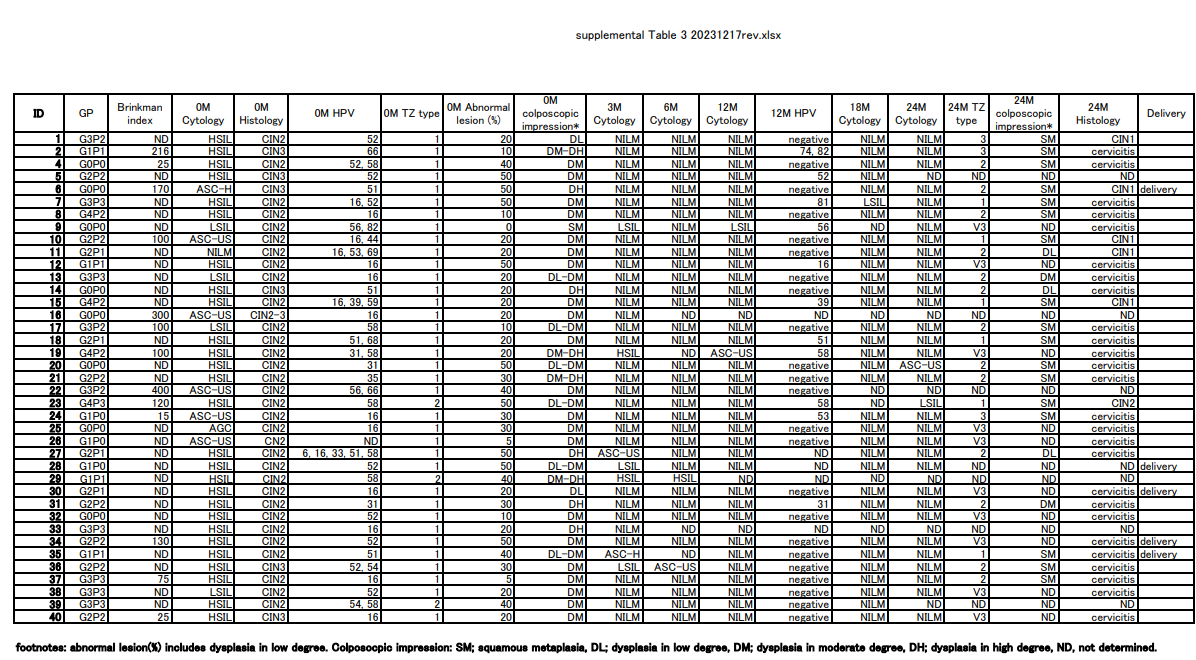

Supplement: Supplementary file 1 — Supplementary Information. [file 41598_2024_53197_MOESM1_ESM.docx]
